# N-Terminal Basic Helix and S120 Phosphorylation Cooperatively Regulate Nuclear Localization of UBE2A/B in Mechanotransduction

**DOI:** 10.3390/biology15181633

**Published:** 2026-09-16

**Authors:** Mingwei Feng, Fumihiko Nakamura

**Affiliations:** School of Pharmaceutical Science and Technology, Tianjin University, 92 Weijin Road, Nankai District, Tianjin 300072, China; fmw_1996@tju.edu.cn

**Keywords:** UBE2A/B, XPO4, phosphorylation, cell density, gene expression

## Abstract

Cells are continuously exposed to mechanical stimuli arising from the extracellular matrix, neighboring cells, and tissue architecture. Through mechanotransduction, these physical cues are converted into intracellular signaling events that regulate cellular behavior and fate. In our previous study, we showed that UBE2A/B localize predominantly in the cytosol when cells are cultured on soft substrates or at high density but translocate to the nucleus on stiff substrates or at low density. This mechanosensitive translocation promotes H2B monoubiquitination and regulates the expression of genes involved in cell growth. However, the mechanism underlying UBE2A/B nuclear localization remains unclear. In the present study, we used mutagenesis to identify regulatory post-translational modifications and define a putative nuclear localization signal (NLS) within UBE2A/B. The phospho-mimetic mutant S120D enhanced UBE2A nuclear localization, whereas substitution of three arginine residues within the predicted NLS (3RA) reduced nuclear localization under low-density conditions. Pharmacological inhibition of cyclin-dependent kinases (CDKs), which are known to phosphorylate S120, suppressed both exogenous and endogenous UBE2A/B nuclear localization and decreased H2B monoubiquitination. Using pull-down assays, we demonstrated that XPO4, a bidirectional nuclear transport receptor, interacts with S120D UBE2A/B but not with wild-type UBE2A/B. Structural modeling suggested that XPO4 interacts with the N-terminal basic helix and phosphorylated S120 residue of UBE2A/B, consistent with our mutational analyses. Moreover, the 3RA mutation abolished the enhanced nuclear localization conferred by S120D. Analysis of public RNA-seq data identified extensive mechanosensitive regulatory programs controlling CDK activity, which align with the nuclear translocation dynamics of UBE2A/B. Together, these findings demonstrate that UBE2A/B nuclear localization is governed by coordinated regulation of its N-terminal basic helix and S120 phosphorylation and identify XPO4 as a potential mediator of its nuclear import.

## Simple Summary

Cells experience physical cues from their environment, which influence gene expression through mechanotransduction. We previously found that Ubiquitin-conjugating enzyme E2 A/B (UBE2A/B) move into the nucleus under stiff or low-density conditions, promoting histone H2B (H2B) monoubiquitination and cell growth-related gene expression, but the underlying mechanism was unknown. Here, we identify key regulatory elements controlling UBE2A/B’s translocation. A phospho-mimetic mutation at S120 (S120D) enhances nuclear localization, while disruption of a basic N-terminal nuclear localization signal (3RA) reduces it. Cyclin-dependent kinase (CDK) inhibition blocks UBE2A/B nuclear entry and decreases H2B ubiquitination. We also show that the nuclear transport receptor Exportin-4 (XPO4) interacts specifically with phosphorylated UBE2A/B and that this interaction depends on both S120 phosphorylation and the nuclear localization signal (NLS) region. Structural modeling supports a cooperative binding mechanism. Together, our results reveal that UBE2A/B nuclear localization is controlled by phosphorylation-dependent signaling and NLS integrity, with XPO4 acting as a potential transport mediator.

## 1. Introduction

Mechanotransduction refers to the process by which living organisms sense and respond to mechanical cues from their environment, including gravity, osmotic pressure, and shear stress [1,2]. A central feature of mechanotransduction is the conversion of mechanical stimuli into biochemical signals. This process plays a critical role in regulating cell growth, differentiation, and proliferation. Mechanotransduction is involved in a wide range of biological processes, including skin repair, tissue regeneration, and angiogenesis, whereas its dysregulation contributes to pathological conditions such as fibrosis and cancer progression by altering cellular behavior, including invasion and metastasis [3].

One well-characterized cellular response closely associated with mechanotransduction is contact inhibition of proliferation (CIP), in which cells cease proliferating upon reaching high density [4]. Although density-dependent signaling and force-dependent signaling (mechanotransduction) are not fully overlapping, accumulating evidence suggests that increased cell crowding is accompanied by substantial mechanical alterations. As cell density rises, cell spreading, cytoskeletal tension, and traction force generation progressively decrease, leading to pronounced changes in intracellular force transmission [5]. A wide array of molecules, ion channels, and signaling pathways—such as Piezo-type mechanosensitive ion channel component 1/2 (PIEZO1/2), the yes associated protein 1 (YAP1)/Hippo pathway, mitogen-activated protein kinase (MAPK) signaling, and integrins—have been identified as key regulators in both CIP and mechanotransduction [6,7,8,9]; nevertheless, the underlying mechanisms remain incompletely understood.

Force- or cell density-dependent nucleocytoplasmic translocation has emerged as a critical mechanism linking mechanical stimuli to gene expression and downstream phenotypic outcomes [10]. To date, more than 30 proteins have been reported to undergo nucleocytoplasmic shuttling in response to mechanical stimulation, including transcription factors and epigenetic regulators [11].

In recent years, mechanically induced epigenetic reprogramming has attracted increasing attention. Epigenetic reprogramming refers to dynamic changes in histone or DNA modifications, such as methylation and acetylation, which regulate chromatin structure and gene expression. These modifications play essential roles in both physiological and pathological processes [12,13,14,15], and their dysregulation can lead to severe outcomes, including tumorigenesis. Epigenetic modifications alter chromatin accessibility; for example, Histone H3 lysine 27 trimethylation (H3K27me3) is associated with condensed chromatin and gene silencing [16], whereas Histone H2B lysine 120 monoubiquitination (H2BK120ub) promotes a more open chromatin state, facilitating RNA polymerase II recruitment and transcriptional initiation and elongation [17,18]. In general, epigenetic modifiers, together with transcription factors, orchestrate global gene expression programs.

Mechanical forces can be transmitted to the nucleus via the linker of nucleoskeleton and cytoskeleton (LINC) complex, directly influencing chromatin architecture [19]. In addition, mechanical cues can modulate the subcellular localization of epigenetic regulators, such as Histone deacetylase 4/5 (HDAC4/5) [20]. Through chromatin remodeling, mechanosensitive epigenetic modifiers act as critical determinants of gene expression and cell fate.

We previously reported that UBE2A/B, E2 ubiquitin-conjugating enzymes responsible for H2BK120ub, translocate to the nucleus in response to changes in substrate stiffness, cell density, and myosin inhibition [21]. We further identified gene expression programs regulated by mechanically induced H2B mono-ubiquitination mediated by UBE2A/B, revealing that these target genes are actively involved in cell proliferation [22,23,24,25]. Therefore, precise subcellular localization of UBE2A/B is essential for their proper function. However, the mechanism governing their translocation remains unclear.

To elucidate the mechanisms governing UBE2A/B nuclear import, we computationally predicted their nuclear localization signals, mapped their post-translational modifications, and performed functional mutagenesis. These approaches revealed that the N-terminal basic helix residues R6, R7, and R8, alongside S120 phosphorylation, act as critical nuclear import determinants. Suppressing the specific kinases that target S120 diminished UBE2A nuclear accumulation and compromised downstream H2B mono-ubiquitination. Using pull-down assays and structural modeling, we identified the bidirectional nuclear transport receptor XPO4 as a likely nuclear transporter for UBE2A/B, noting predicted interactions at amino acid residues R7, K14, and the phosphorylated S120 locus. Strikingly, introducing alanine substitutions at R6, R7, and R8 abolished the hyper-nuclear localization typically driven by the phosphomimetic S120D mutation. Moreover, publicly available RNA-sequencing data revealed that the CDKs responsible for this S120 phosphorylation are under comprehensive mechanosensitive regulation. Collectively, our findings demonstrate that UBE2A/B nuclear translocation is tightly regulated by its N-terminal basic cluster and S120 phosphorylation status, potentially mediated via a mechanosensitive XPO4 transport axis.

## 2. Materials and Methods

### 2.1. Cell Culture

HEK293A cells were purchased from HEK293A cells were purchased from Thermo Fisher Scientific (Waltham, MA, USA; Cat. No. R70507). and cultured on standard tissue culture dishes in Dulbecco’s Modified Eagle Medium (DMEM, VivaCell, Shanghai XP Biomed Ltd., Shanghai, China) supplemented with 10% fetal bovine serum (FBS, VivaCell, Shanghai XP Biomed Ltd., Shanghai, China). and 1% penicillin–streptomycin. Cells were maintained at 37 °C in an incubator with 5% CO_2_.

### 2.2. Plasmid Construction

A human cDNA library was prepared from HEK293A cells. Total RNA was extracted using RNAiso Plus (Takara Bio Inc., Kusatsu, Shiga, Japan, Cat. No. 9108) and reverse-transcribed into cDNA using TransScript First-Strand cDNA Synthesis SuperMix (TransGen Biotech Co., Ltd., Beijing, China; Cat. No. AT301) according to the manufacturer’s instructions. The pcDNA3-Ube2a-HA wild-type (WT) plasmid was generated previously [21]. All Ube2a mutants were constructed based on this plasmid using a Q5 Site-Directed Mutagenesis Kit (New England Biolabs, Inc., Ipswich, MA, USA; Cat. No. E0554S) Ube2a WT and S120D fragments were amplified from HA-tagged plasmids, digested with BamHI and NotI, and ligated into the pcDNA3-SBP vector to generate pcDNA3-Ube2a-SBP WT and S120D constructs. KPNA1, KPNA2, KPNA3, KPNA6, TNPO1 (isoform 2), TNPO2 (isoform 2), KPNB1, and RAD18 were amplified from the HEK293A cDNA library. XPO4 was amplified from pLV-CMV-XPO4 (human)-PGK-Puro (Tianjin Wuyuan Biotechnology Co., Ltd., Tianjin, China; Cat. No. YP35960). All amplified genes were cloned into the pFLAG vector using BamHI and NotI restriction sites, with an N-terminal FLAG tag. Primers used in this study are listed in Supplementary Table S1.

### 2.3. Transfection

For cell density-dependent immunofluorescence experiments, reverse transfection was used. Briefly, PEI transfection complexes were prepared according to the manufacturer’s instructions. Cells were seeded at different densities (400%, 200%, 100%, 50%, and 25%, 1 × 10^5^ cells/cm^2^ = 100% density) by serial dilution, followed by immediate addition of the transfection complexes, and harvested the next day.

For pull-down experiments, cells were transfected using PEI or jetPRIME (Polyplus-transfection S.A., Illkirch, France, Cat. No. 101000046) according to the manufacturer’s instructions. Cells were collected 48 h (PEI) or 24 h (JetPrime) after transfection for further analysis.

### 2.4. Immunofluorescence Microscopy

Cells were plated on a 48-well plate coated with 10 ug/mL fibronectin (Corning Incorporated, Corning, NY, USA; Cat. No. 354008) and cultured overnight, then fixed with 1% formaldehyde in culture medium for 10 min and quenched with 10% glycine for 5 min. Cells were permeabilized with 0.5% Triton X-100 in TBS (50 mM Tris-HCl, pH 7.4, 150 mM NaCl) for 5 min and blocked with blocking buffer (2% BSA in TBS containing 0.1% Triton X-100) for 1 h.

Cells were incubated with primary antibodies diluted in blocking buffer for at least 2 h, washed with TBS-0.1%Tx (TBS containing 0.1% Triton X-100) buffer 3 times, and then incubated with secondary antibodies diluted in blocking buffer for 1 h. After washing, nuclei were stained with Hoechst 33342 (1 µg/mL) for 15 min, washed 3 times, and directly imaged on a tissue culture dish. Images were acquired using an EVOS^®^ FL Auto Imaging System (Thermo Fisher) and processed with ImageJ (NIH version 1.54 g).

### 2.5. Western Blot

Cells were seeded in 60 mm dishes at approximately 50% confluency and incubated overnight. After treatment, cells were washed with PBS 3 times and directly lysed in 1xSDS sample buffer containing beta-mercaptoethanol. Samples were heated at 95 °C for 5 min and separated on a 4–20% Bis-Tris gel (ACE Biotechnology, Changzhou, Jiangsu, China; Cat. No. ET15420LGel). Proteins were transferred to nitrocellulose membranes (GE Healthcare Bio-Sciences AB, Uppsala, Sweden) at 30 V for 1 h. Membranes were blocked with 5% non-fat milk in TBST (20 mM Tris–HCl, pH 7.4, 110 mM NaCl, 5 mM MgCl_2_, 0.1% Tween 20) and incubated with primary antibodies overnight at 4 °C. After washing, membranes were incubated with secondary antibodies for 1 h at room temperature and developed using a chemiluminescent substrate WesternBright ECL (Advansta Inc., San Jose, CA, USA).

### 2.6. Streptavidin-Binding Protein (SBP) Pulldown Assay

Cells were seeded in 60 mm dishes at approximately 50% confluency and incubated overnight. Cells were then transfected with pcDNA-Ube2a-SBP [26] or pFLAG-importin plasmids using PEI or JetPrime (Polyplus) and incubated for 48 h (PEI) or 24 h (JetPrime). For phosphatase inhibitor treatment, 50× Phosphatase Inhibitor Cocktail C (Santa Cruz Biotechnology, Inc., Dallas, TX, USA; sc-45065) or DMSO (Santa Cruz Biotechnology, Inc., Dallas, TX, USA; sc-358801) was added to the culture medium and incubated for 30 min before cell harvest. Following treatment, cells were washed three times with PBS and lysed in 200 μL TTBS lysis buffer (50 mM Tris-HCl, pH 7.4, 150 mM NaCl, 1% Triton X-100, 1 mM EGTA, and 1 mM β-mercaptoethanol) containing a Protease inhibitor cocktail (100×, Beyotime Biotechnology, Shanghai, China; Cat. No. P1006) on ice for 10 min. For cells treated with phosphatase inhibitor cocktail C, Phosphatase inhibitor cocktail II (100×, MedChemExpress, Monmouth Junction, NJ, USA; Cat. No. HY-K0022) was additionally added to the TTBS lysis buffer prior to lysis. Following lysis, samples were centrifuged at 15,000 rpm for 10 min, and the supernatants were transferred to new 1.7 mL tubes. Supernatants containing UBE2A-SBP and FLAG-XPO4 were mixed and incubated for 2 h, followed by incubation with 40 μL NHS-streptavidin beads overnight. The beads were then washed four times with TTBS, and bound proteins were eluted with 10 μL of 5× SDS sample buffer by heating at 95 °C for 5 min. Eluted proteins were analyzed by SDS-PAGE.

### 2.7. Antibodies

Detailed information on the antibodies used in this study, including their sources, catalog numbers, and previously reported applications, is provided in Table S2 [27,28,29,30,31,32,33,34,35,36,37,38,39].

### 2.8. Computational Prediction of NLS and Post-Translational Modifications (PTMs)

The NLS of UBE2A was predicted using NLSExplorer [40], NLS Mapper [41], and NLStradamus [42], respectively. The post-translational modification map of UBE2A was obtained from PhosphoSitePlus [43].

### 2.9. AlphaFold 3 Prediction

The complex structures of XPO4–UBE2A and KPNA1–UBE2A were predicted using AlphaFold 3 via the AlphaFold Server (https://alphafoldserver.com/, accessed on 14 April 2026). Amino acid sequences of the proteins of interest were obtained from the UniProt database and submitted to AlphaFold 3 as separate input chains for complex structure prediction. Predictions were performed using the default settings, with no additional structural relaxation or refinement applied after prediction. Structural images were generated using either the built-in visualization tools of the AlphaFold Server or PyMOL (3.0.3) based on the predicted structures.

### 2.10. RNA-Sequencing

Public RNA-seq datasets were obtained from GSE207896 (DMSO vs. blebbistatin) [44], GSE249290 (A375 density) [45], and GSM2701559 (substrate stiffness) [46]. Analysis of differentially expressed genes was conducted using iDEP (http://bioinformatics.sdstate.edu/idep/ (accessed on 14 April 2026)). Differential expression analysis was performed using iDEP. Heatmaps were generated based on Z-score normalization using Excel.

### 2.11. Data Analysis

Statistical significance was evaluated using unpaired *t*-tests or one-/two-way ANOVA in GraphPad Prism 9.5.0. Statistical significance was defined as * *p* < 0.05, ** *p* < 0.01, *** *p* < 0.001, **** *p* < 0.0001. Image analysis was performed in a blind manner. Raw data are provided in Supplementary Table S3.

### 2.12. AI Statement

During the preparation of this manuscript, the authors used ChatGPT (GPT-5.6 Luna) to improve the language and readability of the manuscript. The authors reviewed and edited all AI-assisted content and take full responsibility for the final version of the manuscript.

## 3. Results

### 3.1. Differential Spatial Dynamics of UBE2A/B and YAP1 Under Varying Cell Density Conditions

YAP1 is known to translocate to the cytoplasm in response to increased cell density [8]. Similarly, we previously demonstrated that UBE2A/B translocate to the cytosol at high cell density [21]. However, the threshold cell density at which YAP1 and UBE2A/B undergo translocation remains unclear. To address this, HEK293A cells were used to examine density-dependent nucleocytoplasmic shuttling of YAP1 and UBE2A/B. Cells were seeded at densities ranging from 25% to 400% (with 100% defined as 1 × 10^5^ cells/cm^2^ [47]) using serial dilution and stained with anti-YAP1 and anti-UBE2A/B antibodies.

**Figure 1:**
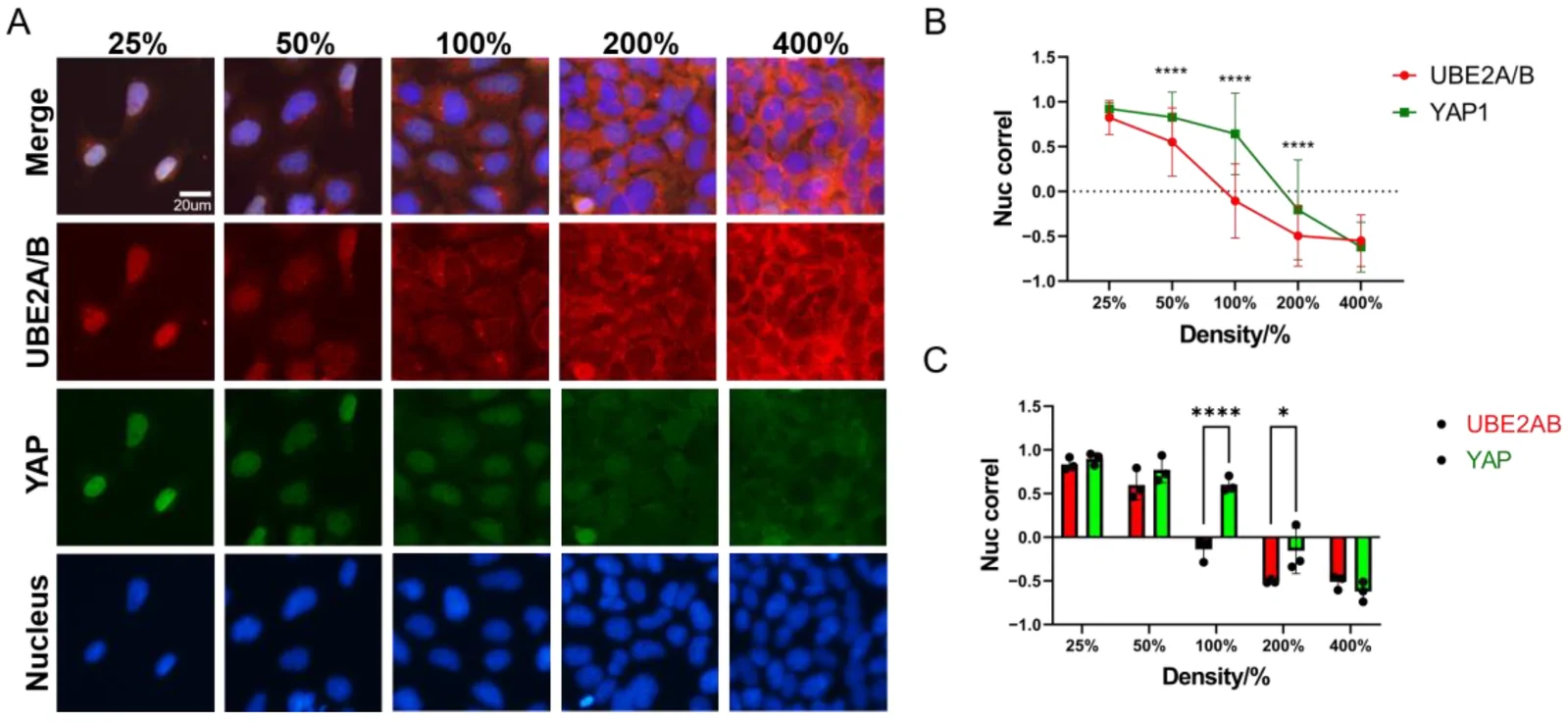
Divergent nucleocytoplasmic shuttling dynamics of UBE2A/B and YAP1 in response to cell density. (**A**) HEK293A cells were cultured at densities ranging from 25% to 400%, with 100% corresponding to 1 × 10^5^ cells/cm^2^. Cells were stained with a rabbit anti-UBE2A/B antibody, detected with a goat anti-rabbit IgG secondary antibody conjugated to Alexa Fluor 594 (red), and a mouse anti-YAP1 antibody, detected with a goat anti-mouse IgG secondary antibody conjugated to Alexa Fluor 488 (green). Nuclei were stained with Hoechst 33342 (blue). Scale bar, 20 μm. (**B**) Grouped line plot showing changes in the mean correlation index of UBE2A/B (red) and YAP1 (green) across different cell densities. YAP1 exhibited a rapid shift from predominantly nuclear to cytosolic localization between 100% and 200% density, whereas UBE2A/B showed a gradual redistribution with increasing cell density. Error bars represent SD. Statistical significance was determined using two-way ANOVA with Tukey’s multiple comparisons test (n = 92–109 cells per condition, N = 3 independent experiments; **** *p* < 0.0001). Each dot represents the mean value obtained from the analyzed cells, and error bars represent SD. (**C**) Bar graph showing changes in the mean correlation index of UBE2A/B (red) and YAP1 (green) with the nucleus (blue) across different cell densities. Statistical significance was determined using two-way ANOVA with Šídák’s multiple comparisons test (N = 3 independent experiments; * *p* < 0.05, **** *p* < 0.0001). Each dot represents an independent biological replicate.

The nuclear versus cytoplasmic distribution of the protein of interest (POI) was quantified by calculating a correlation index (CI) between the POI signal and the Hoechst signal along a line spanning both the nucleus and cytoplasm of individual cells. The CI ranges from −1 to 1, with values close to 1 indicating nuclear localization and values close to −1 indicating cytoplasmic localization [21]. Representative examples of YAP1 CI calculation are shown in Figure S1, demonstrating the reliability of this method for quantifying subcellular distribution. Quantitative analysis of YAP1 and UBE2A/B staining (Figure 1B,C) revealed distinct translocation patterns between these proteins. Although both YAP1 and UBE2A/B exhibited predominantly cytosolic localization at 400% density, their transition dynamics differed markedly. YAP1 was primarily nuclear at 25–100% density and underwent a rapid shift to the cytoplasm when cell density reached 200%. In contrast, UBE2A/B displayed a gradual and continuous redistribution from the nucleus to the cytosol as cell density increased, without an apparent threshold. These results suggest that UBE2A/B and YAP1 are regulated by distinct mechanisms governing nucleocytoplasmic shuttling.

### 3.2. Identification of the N-Terminal Basic Helix and S120 as Essential Regulators of UBE2A/B Nuclear Trafficking

**Figure 2:**
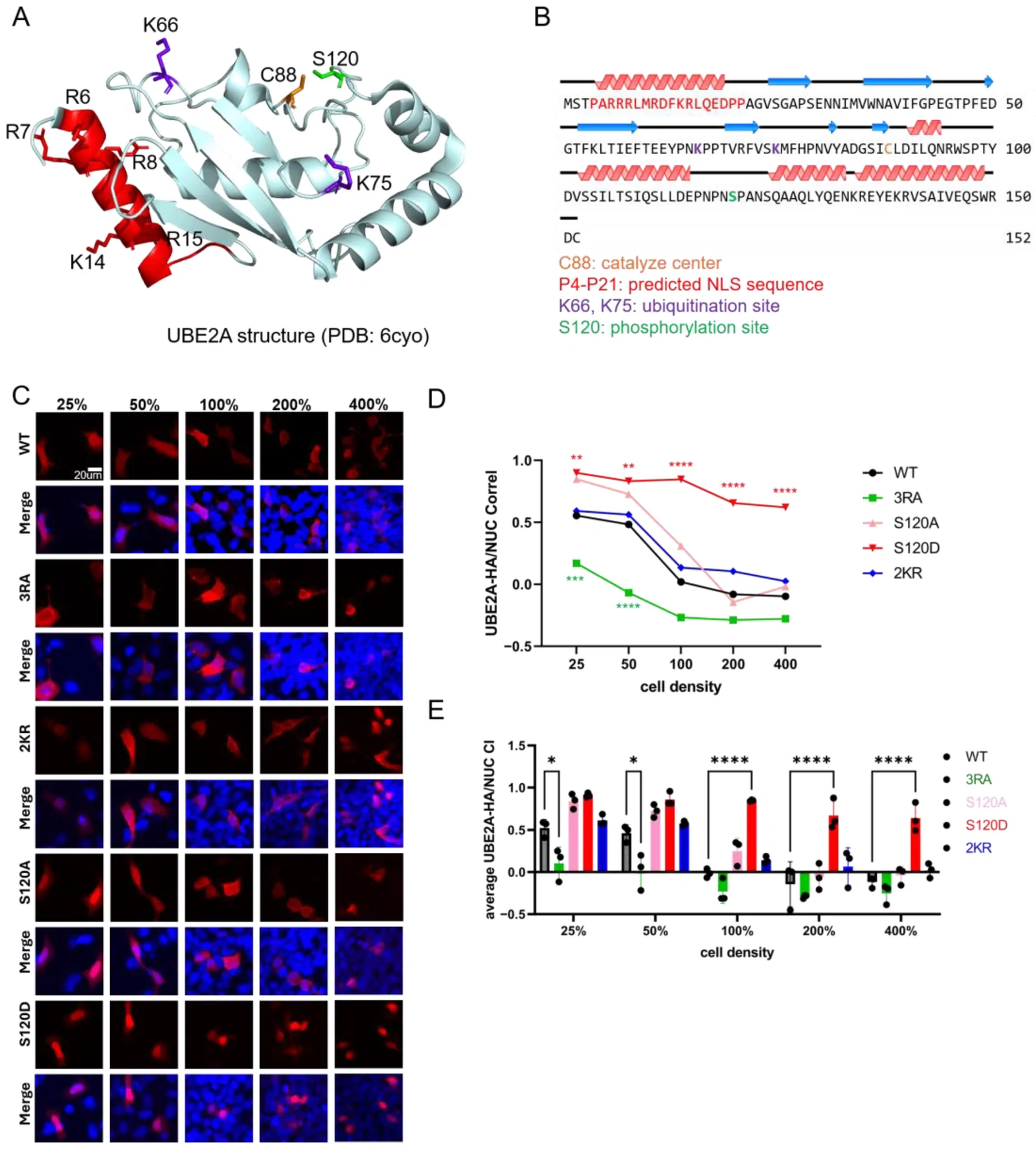
The N-terminal basic helix and S120 regulate UBE2A/B nucleocytoplasmic trafficking. (**A**) Crystal structure of UBE2A (PDB: 6CYO). (**B**) Amino acid sequence of UBE2A (152 residues). The catalytic center C88 is highlighted in orange in both the structure (**A**) and sequence (**B**). The predicted NLS is indicated in red, ubiquitination sites in purple, and phosphorylation sites in green. (**C**) Density-dependent nucleocytoplasmic shuttling of UBE2A-HA WT and mutants. HA-tagged WT, R6A/R7A/R8A (3RA), K66R/K75R (2KR), S120A, and S120D constructs were transfected into HEK293A cells at densities ranging from 25% to 400%. Cells were fixed 24 h post-transfection and stained with a rabbit polyclonal anti-HA antibody (detected with goat anti-rabbit IgG secondary antibody conjugated to Alexa Fluor 594, red), and nuclei were stained with Hoechst 33342 (blue). (**D**) Grouped line plot summarizing the correlation index (CI) between HA signal intensity and Hoechst signal intensity for each construct. The S120D mutant exhibited stronger nuclear localization across all cell densities, whereas the 3RA mutant showed reduced nuclear localization at low cell densities compared with WT. Each dot represents the mean value obtained from the analyzed cells, and error bars represent SD. Statistical analysis was performed using two-way ANOVA with Dunnett’s multiple comparisons test (n = 13–62 cells per condition, N = 3 independent experiments; ** *p* < 0.01, *** *p* < 0.001, **** *p* < 0.0001). (**E**) Bar graph showing changes in the mean correlation index of each construct across different cell densities. Statistical analysis was performed using two-way ANOVA with Tukey’s multiple comparisons test (N = 3 independent experiments; * *p* < 0.05, **** *p* < 0.0001). Each dot represents an independent biological replicate.

Next, we sought to investigate the regulatory mechanisms underlying UBE2A/B nucleocytoplasmic shuttling. In general, nucleocytoplasmic transport is mediated by post-translational modifications (PTMs) and NLSs [48,49]. To identify potential regulatory sites, we queried the PhosphoSitePlus database (https://www.phosphosite.org/) and found that UBE2A contains three phosphorylation sites (S120, S142, and S148), two ubiquitination sites (K66 and K75), and one acetylation site (K66) (Figure S2A). Based on previous studies, among the reported phosphorylation sites, only S120 has been experimentally validated in vivo [50]. Therefore, we focused on S120 for further analysis. To predict potential NLS regions, multiple publicly available prediction tools were used [40,41,42]. All predictions consistently identified a putative NLS within the N-terminal region (amino acids 4–40) of UBE2A (Figure S2B–D). Given that NLS motifs are typically enriched in basic residues, we focused on residues R6, R7, and R8 as candidate functional sites. Structural information of UBE2A was also examined to ensure that these residues are surface-exposed and accessible for interaction. To evaluate the functional roles of PTMs, we generated K66R/K75R (2KR, ubiquitination-deficient), S120A (phospho-deficient), and S120D (phospho-mimetic) mutants. To assess the role of the predicted NLS, we generated an R6A/R7A/R8A (3RA) mutant (Figure 2A,B).

HEK293A cells were transfected with UBE2A-HA constructs carrying the indicated mutations and cultured at densities ranging from 25% to 400%. Subcellular localization was quantified using the HA nuclear correlation index. Results were compared to wild-type controls using two-way ANOVA. Quantitative analysis showed that the 3RA mutant exhibited significantly reduced nuclear localization at low cell density. In contrast, although the S120A mutant showed no significant difference compared to the wild type, the S120D mutant displayed a significant increase in nuclear localization across all tested cell densities (Figure 2C,E). These results suggest that both the NLS and S120 phosphorylation contribute to the regulation of UBE2A/B nucleocytoplasmic shuttling.

### 3.3. Inhibition of CDKs Suppresses Nuclear Localization of UBE2A/B and H2B Mono-Ubiquitination

**Figure 3:**
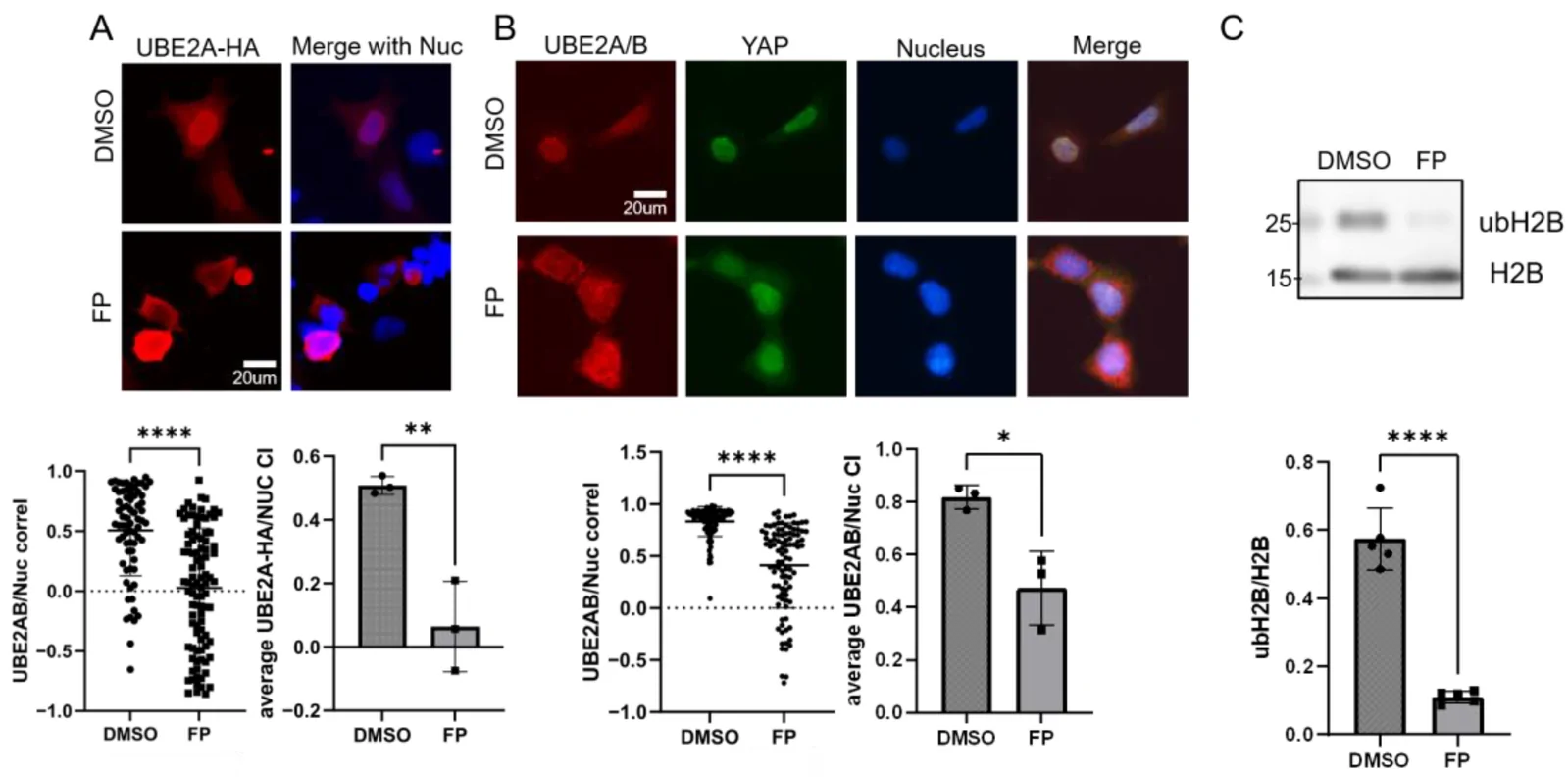
Suppression of UBE2A/B nuclear localization and H2B mono-ubiquitination via pharmacological CDK inhibition. (**A**) Inhibition of CDKs promotes cytosolic localization of exogenously expressed UBE2A. HEK293A cells were transfected with wild-type UBE2A-HA. After 24 h, cells were treated with 0.5 μM flavopiridol (FP) or DMSO for 6 h, then stained with a rabbit polyclonal anti-HA antibody (detected with goat anti-rabbit IgG secondary antibody conjugated to Alexa Fluor 594, red), and nuclei were stained with Hoechst 33342 (blue). Quantification of the UBE2A nuclear correlation index shows a significant reduction after FP treatment, indicating cytosolic translocation of UBE2A-HA (statistical significance was determined using an unpaired Student’s *t*-test, ** *p* < 0.01, **** *p* < 0.0001.). In the bottom-left panel, each dot represents an individual cell measurement (n = 72–95 cells, N = 3 independent experiments); in the bottom-right panel, each dot represents the mean of an independent biological replicate (N = 3). (**B**) Inhibition of CDK2/9 promotes cytosolic localization of endogenous UBE2A/B. HEK293A cells were plated in 48-well plates, cultured for 24 h, and treated with 0.5 μM FP or DMSO for 6 h. Cells were stained with rabbit anti-UBE2A/B antibody (detected with Alexa Fluor 594, red), mouse anti-YAP1 antibody (detected with Alexa Fluor 488, green), and nuclei with Hoechst 33342 (blue). Quantitative analysis demonstrates cytosolic translocation of endogenous UBE2A/B upon FP treatment (unpaired Student’s *t*-test, * *p* < 0.05, **** *p* < 0.0001.). In the bottom-left panel, each dot represents an individual cell measurement (n = 98–104 cells, N = 3 independent experiments); in the bottom-right panel, each dot represents the mean of an independent biological replicate (N = 3). (**C**) CDK inhibition reduces H2B K120 mono-ubiquitination. HEK293A cells were plated in 35 mm dishes, cultured for 24 h, and treated with 0.5 μM FP or DMSO for 6 h. Levels of total H2B and mono-ubiquitinated H2B were analyzed by Western blot using anti-H2B and anti-K120ub antibodies. Quantification shows a significant decrease in H2BK120ub upon FP treatment (unpaired Student’s *t*-test, N = 5, **** *p* < 0.0001.).

To further validate the role of phosphorylation in regulating UBE2A/B subcellular localization, we inhibited upstream kinases targeting the S120 site. Previous studies have identified CDK2 and CDK9 as potential kinases that phosphorylate UBE2A/B at S120 [50,51]. However, these findings are not fully consistent and may depend on cell type or cell cycle context. To ensure effective inhibition of upstream kinase activity, we employed flavopiridol (FP), a broad-spectrum cyclin-dependent kinase (CDK) inhibitor, in subsequent experiments. Immunofluorescence analysis showed that inhibition of CDKs significantly reduced the nuclear localization of both exogenous UBE2A-HA and endogenous UBE2A/B (Figure 3A,B). In addition, Western blot analysis demonstrated that flavopiridol treatment decreased the level of H2BK120 mono-ubiquitination, a key downstream target of UBE2A/B (Figure 3C). These findings suggest that CDK-dependent phosphorylation is indispensable for the nuclear translocation and subsequent catalytic activity of UBE2A/B toward H2BK120.

### 3.4. Selective Binding of XPO4 to Phosphorylated UBE2A/B Mimics over Wild-Type Controls

**Figure 4:**
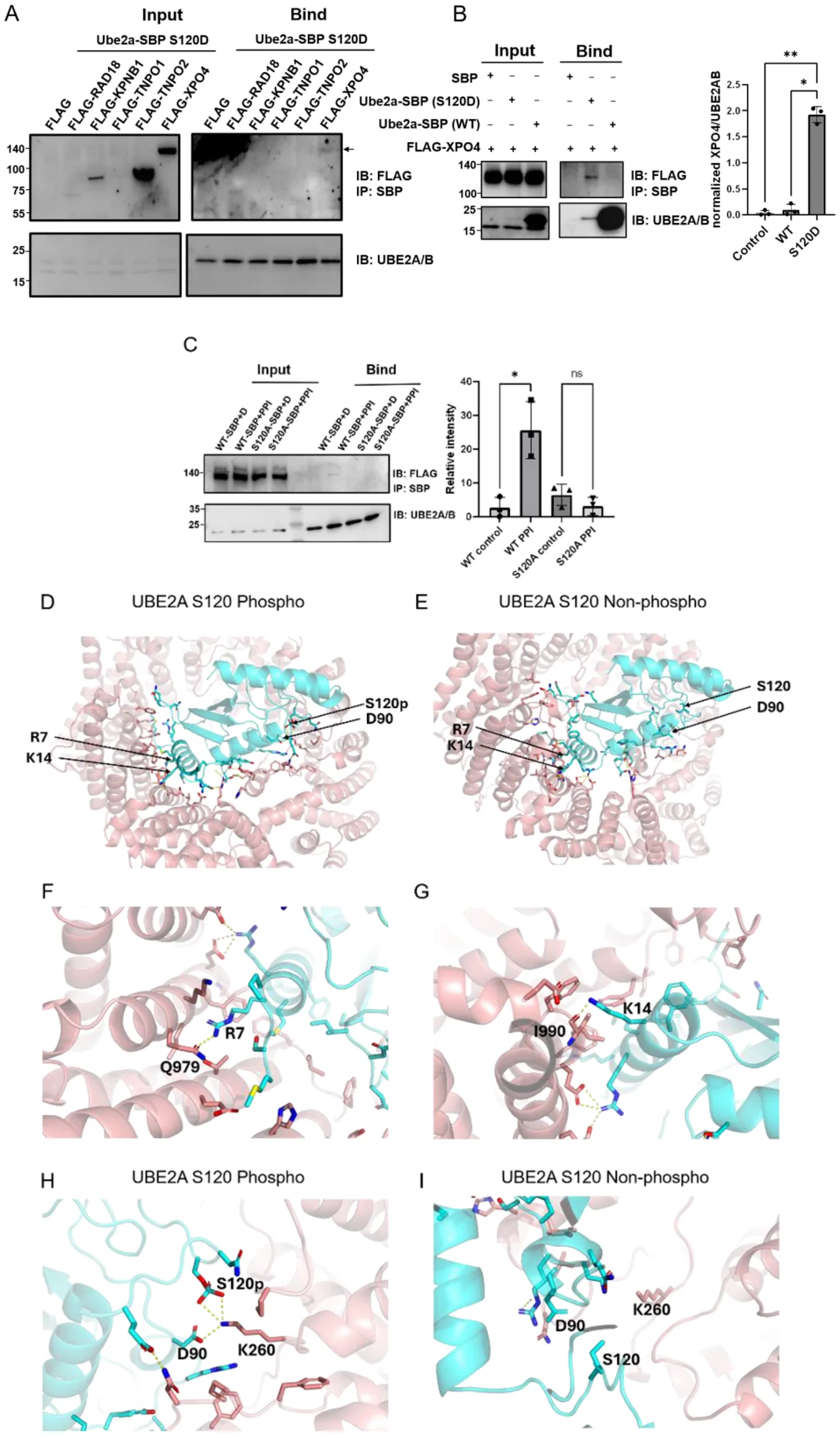
Identification of XPO4 as a potential nuclear importer of phosphorylated UBE2A/B. (**A**) Interaction of XPO4 with phospho-mimetic UBE2A-SBP. FLAG-tagged RAD18 (positive control), KPNB1, TNPO1, TNPO2, and XPO4, together with SBP-tagged UBE2A-SBP S120D, were expressed in HEK293A cells for 24 h. Cell lysates were incubated with NHS-streptavidin beads for pull-down. Bound FLAG-tagged transport proteins were detected by Western blot using an anti-FLAG antibody, and UBE2A-SBP was detected using an anti-UBE2A/B antibody. (**B**) XPO4 preferentially interacts with phospho-mimetic UBE2A-SBP. FLAG-tagged XPO4 and SBP-tagged wild-type or S120D UBE2A-SBP were co-expressed in HEK293A cells. Cell lysates were subjected to SBP pull-down using NHS-streptavidin beads. Bound FLAG-XPO4 was detected by Western blot using an anti-FLAG antibody. Quantification shows a significant increase in interaction with the S120D mutant compared with WT and control (N = 3; one-way ANOVA, * *p* < 0.05, ** *p* < 0.01). (**C**) Phosphatase inhibition enhances the interaction between XPO4 and UBE2A. FLAG-tagged XPO4 and SBP-tagged wild-type or S120A UBE2A were expressed in HEK293A cells. After 24 h, cells were treated with phosphatase inhibitor cocktail C (Santa Cruz, sc-45065) or DMSO for 30 min. For the phosphatase inhibitor treatment group, an additional phosphatase inhibitor cocktail (MCE, HY-K0021) was added to the TTBS lysis buffer during cell lysis and incubation. Bound FLAG-XPO4 was detected by Western blotting using an anti-FLAG antibody (N = 3; two-way ANOVA, Tukey’s multiple comparisons test, * *p* < 0.05, ns: no significant difference.). (**D**,**E**) Predicted structure of the UBE2A-XPO4 complex generated using AlphaFold 3. Cyan, UBE2A; salmon, XPO4. The interface residues are opaque, while the rest are transparent. (**F**,**G**) Magnified views showing the side-chain interactions between the N-terminal basic helix of UBE2A and XPO4. Panel D shows the interaction between UBE2A Arg7 and XPO4 Gln979. Panel E shows the interaction between UBE2A Lys14 and XPO4 Ile990. (**H**,**I**) Structural comparison of phosphorylated and non-phosphorylated UBE2A in complex with XPO4. A potential interaction involving UBE2A pSer120 and Asp90 with XPO4 Lys260 is observed specifically in the phosphorylated UBE2A-XPO4 complex but not in the non-phosphorylated complex.

Next, we sought to determine the mechanism underlying its nuclear import. Active nuclear transport typically requires transport receptors, particularly the importin-α (KPNA) and importin-β (KPNB) families. In the canonical nuclear import pathway, cargo proteins bind to KPNA via basic NLS motifs, and the cargo–KPNA complex subsequently associates with KPNB1 for translocation into the nucleus, followed by Ran-GTP-dependent dissociation [52]. To assess whether UBE2A/B translocation depends on the canonical KPNA/KPNB1 pathway, we performed SBP pull-down assays. FLAG-tagged KPNA1/2/3/6 or wild-type UBE2A-SBP were expressed in HEK293A cells, and cell lysates were mixed and incubated with streptavidin beads. Because lysates were diluted in TTBS buffer, the concentration of Ran-GTP is expected to be low, mimicking cytoplasmic conditions that favor cargo–importin interaction [53,54]. However, no detectable enrichment of UBE2A with KPNA1/2/3/6 was observed in the pulldown assay under the conditions tested (Figure S3A). A similar result was observed using the UBE2A-SBP S120D mutant (Figure S3B). These findings do not support a major role for the canonical KPNA/KPNB1 pathway in mediating UBE2A/B nuclear import under the conditions examined. In addition to the canonical pathway, certain cargo proteins can be transported directly by members of the KPNB family. Previous proteomic studies have identified candidate cargo proteins for multiple importins and biportins (a bidirectional nuclear transport receptor) [55]. Intriguingly, bioinformatic interrogation of these datasets highlighted UBE2A as a candidate interactor for several nuclear transport receptors. To experimentally validate these targets, we cloned and expressed FLAG-tagged variants of three top candidates—TNPO1, TNPO2, and XPO4—and performed streptavidin-binding peptide (SBP) pull-down assays utilizing a phosphomimetic UBE2A-SBP S120D mutant. Strikingly, only XPO4 demonstrated a robust and reproducible interaction with UBE2A (Figure 4A), implicating XPO4 as the primary nuclear transporter mediating UBE2A/B trafficking.

**Figure 5:**
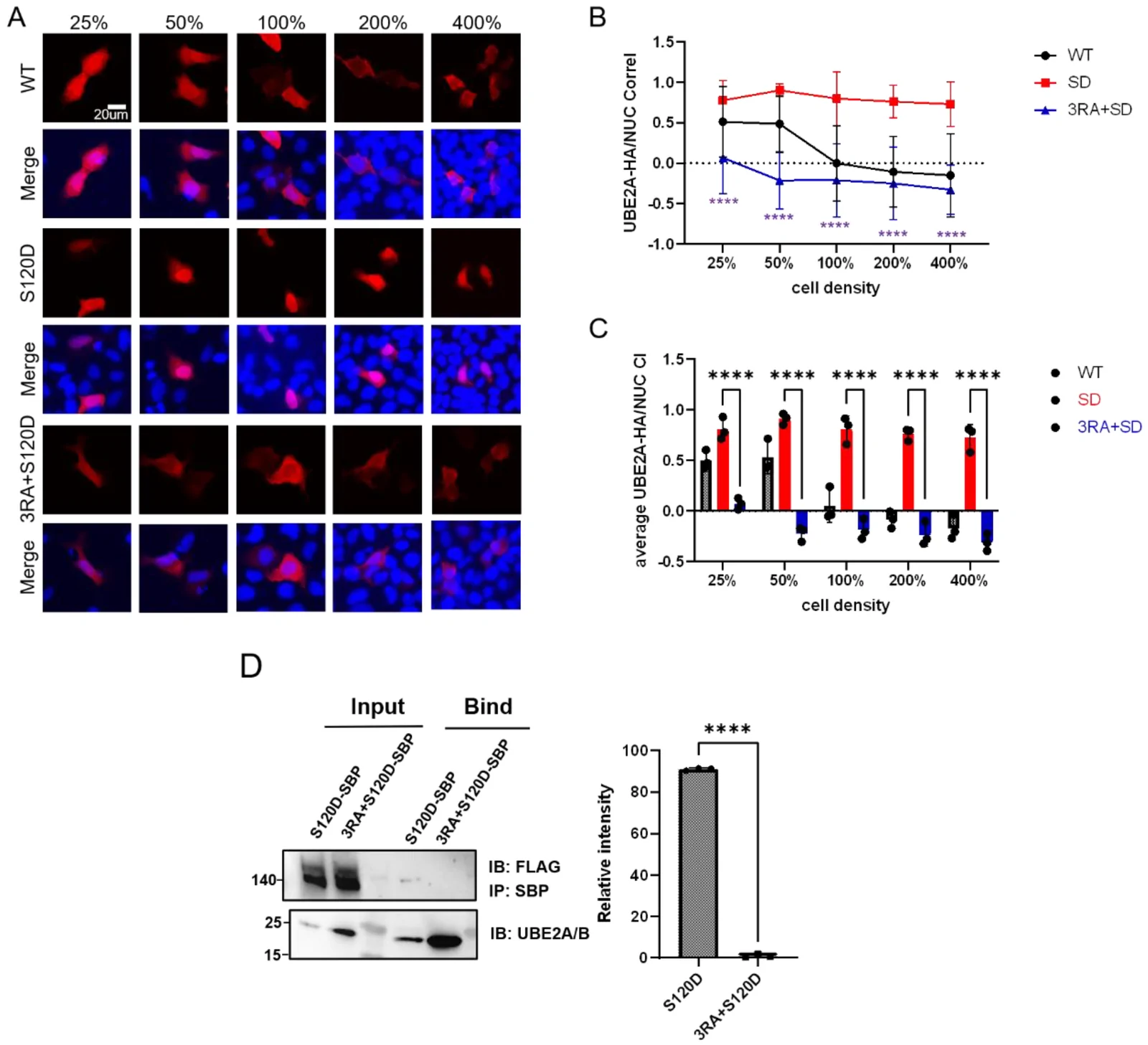
Alanine mutations of the N-terminal basic cluster abrogate the enhanced nuclear localization of UBE2A/B S120D. (**A**) Density-dependent nucleocytoplasmic shuttling of UBE2A-HA WT, S120D, and 3RA+S120D mutants. HA-tagged WT, S120D, and 3RA+S120D constructs were transfected into HEK293A cells. Fixation and staining were performed as described in Figure 2C. (**B**) Grouped line plot showing the HA nuclear correlation index (CI) in cells expressing the indicated constructs. The 3RA+S120D mutant exhibited increased cytosolic localization across all cell densities compared with the S120D mutant. Statistical analysis was performed using two-way ANOVA with Tukey’s multiple comparisons test (n = 18–35 cells per condition, N = 3 independent experiments; **** *p* < 0.0001). (**C**) Bar graph showing changes in the mean correlation index of each construct across different cell densities. Statistical analysis was performed using two-way ANOVA with Tukey’s multiple comparisons test (N = 3 independent experiments; **** *p* < 0.0001). Each dot represents an independent biological replicate. (**D**) Addition of the 3RA mutation to the UBE2A-S120D mutant abolishes the interaction between UBE2A and XPO4. FLAG-tagged XPO4 and SBP-tagged S120D or 3RA+S120D mutant UBE2A were expressed in HEK293A cells. Bound FLAG-XPO4 was detected by Western blotting using an anti-FLAG antibody. Quantification showed a significant reduction in the interaction of XPO4 with the 3RA+S120D mutant compared with the S120D mutant (N = 3; unpaired Student’s *t*-test).

To further investigate the role of phosphorylation, we examined the interaction between FLAG-XPO4 and either WT or S120D UBE2A-SBP. Notably, XPO4 interacted only with the S120D mutant, but not with WT (Figure 4B), suggesting that the interaction is phosphorylation-dependent. We therefore selected phosphatase inhibitor cocktail C, which broadly inhibits serine/threonine phosphatases, including phosphatases that can antagonize CDK-mediated phosphorylation. Consistent with our hypothesis, treatment with the phosphatase inhibitor enhanced the interaction between wild-type UBE2A-SBP and FLAG-XPO4 (Figure 4C). To gain structural insight into this interaction, we modeled the UBE2A–XPO4 complex using AlphaFold 3 (Figure 4D,E). The predicted structure suggests that UBE2A interacts with XPO4 through its N-terminal region. Specifically, Arg7 of UBE2A forms contacts with Gln979 of XPO4 (Figure 4F), whereas Lys14 of UBE2A interacts with Ile990 of XPO4 (Figure 4G). In addition, phosphorylation at S120 may introduce an additional interaction interface, with phosphorylated Ser120 and Asp90 of UBE2A positioned to interact with Lys260 of XPO4 (Figure 4H,I). Although the confidence metrics of the predicted complex were relatively low (Figure S4), the predicted interaction interface is consistent with our biochemical analysis. Notably, AlphaFold 3 predicted a distinct interaction pattern for the KPNA1–UBE2A complex, in which the N-terminal helix of UBE2A is positioned away from the protein–protein interface (Figure S5). This is consistent with our pulldown assay results, in which no detectable enrichment of UBE2A with KPNA1 was observed under the conditions tested and underscores the specificity of the predicted XPO4–UBE2A interaction. These structural predictions are also consistent with the phenotypes observed for the 3RA and S120D mutants, further supporting a phosphorylation-dependent interaction between UBE2A and XPO4. To further evaluate the combined contribution of the N-terminal basic residues and phosphorylation, we generated a 3RA-S120D double mutant. Immunofluorescence analysis showed that introduction of the 3RA mutation into the S120D background reversed the enhanced nuclear localization of the S120D mutant (Figure 5A). Two-way ANOVA analysis confirmed a significant decrease in nuclear localization of UBE2A-HA in the 3RA+S120D mutant compared with S120D alone (Figure 5B,C). The pulldown assay further showed that the interaction between UBE2A-S120D and XPO4 was reduced in the presence of the 3RA mutant (Figure 5D). Together, these findings demonstrate that UBE2A/B nuclear localization is coordinately governed by its N-terminal basic cluster and S120 phosphorylation status, while establishing XPO4 as a candidate nuclear transport receptor mediating this process.

### 3.5. Mechanical Cues Govern the Activity of Upstream CDKs That Phosphorylate UBE2A/B

**Figure 6:**
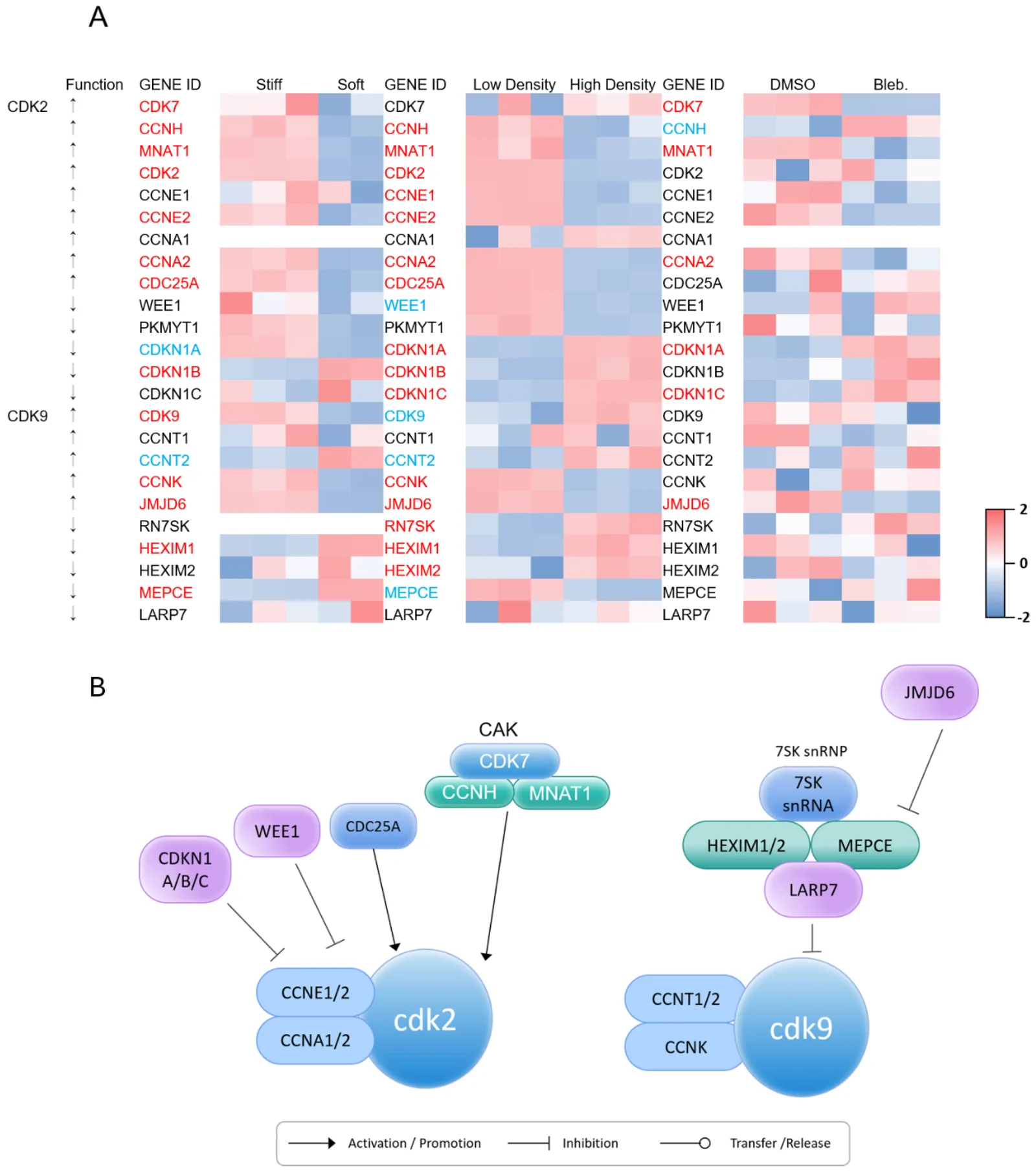
Upstream kinases of UBE2A/B are regulated by mechanical cues. (**A**) Heatmaps showing expression of regulatory genes associated with CDK2/CDK9 in cells cultured under different mechanical conditions, including varying substrate stiffness, cell density, and myosin inhibition. Based on the working hypothesis, genes that promote CDK2/9 activity are expected to be upregulated under stiff substrate, low-density, or DMSO-treated conditions. Red labels indicate genes consistent with this hypothesis, whereas blue labels indicate the opposite trend. Across the three conditions, the proportions of genes consistent with the hypothesis were 13/21, 15/23, and 6/22, respectively, while genes showing the opposite trends were 2/21, 4/23, and 1/22 (p_adj_ < 0.05). These results suggest that CDK2/CDK9 activity is generally upregulated under stiff substrate, low-density, or DMSO-treated conditions. (**B**) Schematic diagram of regulatory pathways of CDK2 and CDK9. CDK2 forms active complexes with Cyclin E1/E2 or Cyclin A1/A2. CDK9 forms complexes with Cyclin T1/T2 or Cyclin K. The CAK (CDK-activating kinase) complex activates CDK2 via phosphorylation, whereas Cyclin-dependent kinase inhibitor 1/1B/1C (CDKN1A/B/C, p21/p27/p57) inhibits CDK2 activity. WEE1 suppresses CDK2 activity by inhibitory phosphorylation, while M-phase inducer phosphatase 1 (CDC25A) reverses this effect by dephosphorylation. CDK9 is negatively regulated by the7SK small nuclear ribonucleoprotein (7SK snRNP) complex, which consists of 7SK snRNA, hexamethylene bis-acetamide-inducible protein 1/2 (HEXIM1/2), 7SK snRNA methylphosphate capping enzyme (MEPCE), and La-related protein 7 (LARP7). JMJD6 can cleave MEPCE, releasing CDK9 from the inhibitory complex.

Given that CDK2 and CDK9 have been implicated as candidate UBE2A/B kinases [47,48], we hypothesized that the expression or catalytic activity of these enzymes might be mechanosensitive. Because the activities of CDK2 and CDK9 are regulated by multiple upstream activators and inhibitors, we reasoned that coordinated transcriptional changes across these regulatory components could provide indirect evidence for altered kinase activity under different mechanical conditions. To test this hypothesis, we interrogated publicly available transcriptomic datasets derived from cells subjected to various mechanical perturbations, including cultivation of hepatic stellate cells on soft versus stiff substrates [46], cultivation of A375 cells with variations in seeding density [45], and pharmacological inhibition of myosin in mouse tendon fibroblasts [44] (Figure 6).

The expression levels of key regulatory genes were visualized as heatmaps (Figure 6A). Genes were classified according to whether their reported functions would be expected to enhance or suppress CDK2/CDK9 activity. We then evaluated whether their transcriptional changes under different mechanical conditions were consistent with the predicted direction of kinase activation. Despite differences in dataset composition and analysis pipelines, consistent trends were observed. Among the selected genes, 13 out of 21 showed expression changes consistent with known pathways illustrated in Figure 6B, whereas 2 showed the opposite trend. Similarly, 15 out of 23 genes supported increased kinase activity in low-density conditions, with 4 showing opposite changes. In the blebbistatin dataset, 6 out of 22 genes supported increased kinase activity under DMSO control conditions, compared with 1 showing the opposite trend.

## 4. Discussion

**Figure 7:**
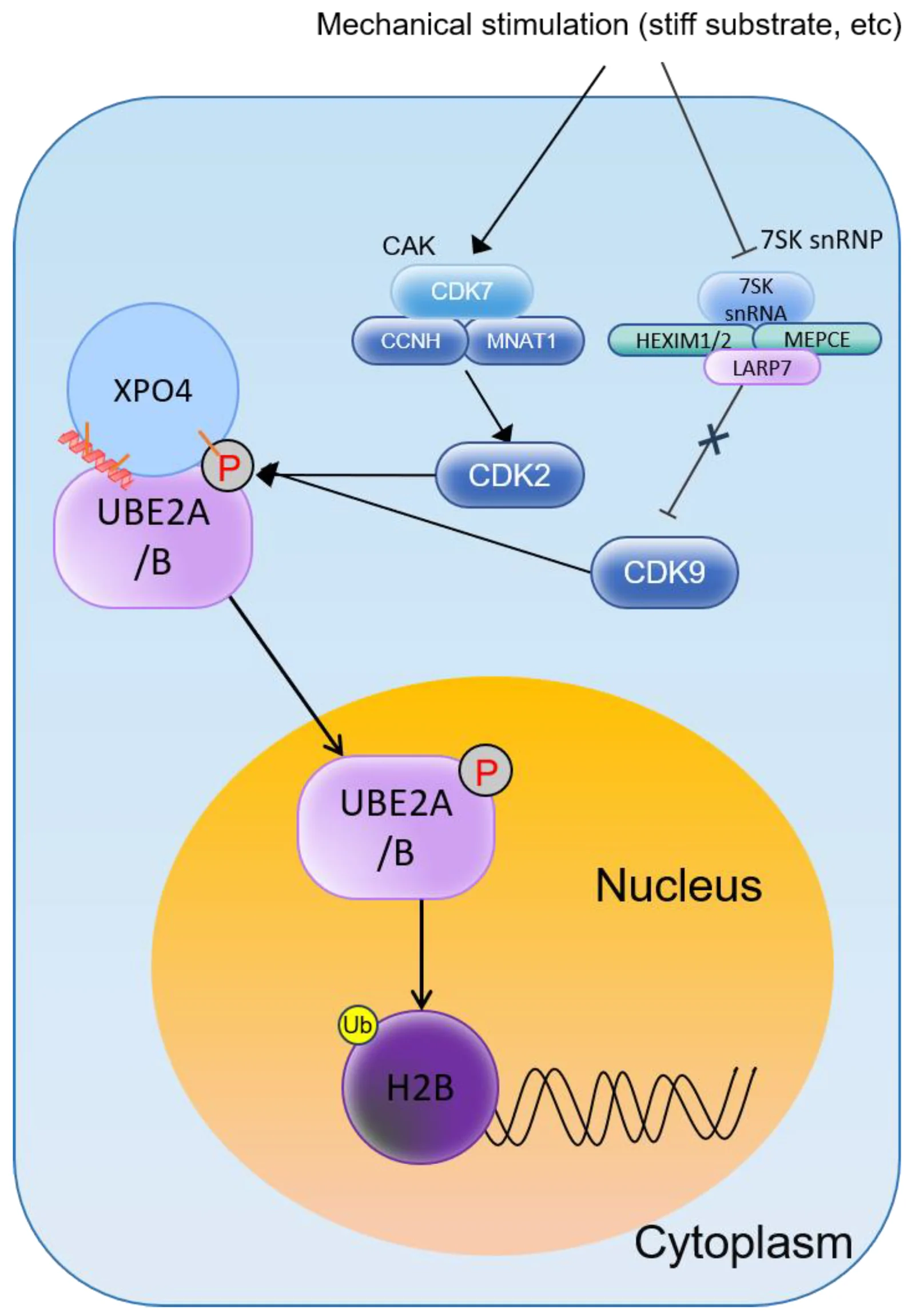
Mechanical stimulation-mediated phosphorylation promotes nuclear translocation of UBE2A/B and H2B mono-ubiquitination. Schematic model illustrating the proposed mechanism. Under conditions such as low cell density or a stiff substrate, upstream regulators of CDK2 and CDK9, including the CAK complex and 7SK snRNP, are modulated, leading to increased CDK2/9 activity and enhanced phosphorylation of UBE2A/B. Phosphorylated UBE2A/B interacts with XPO4 via its N-terminal basic residues and phosphorylation site, facilitating nuclear import. Accumulation of UBE2A/B in the nucleus promotes downstream processes, including H2B K120 mono-ubiquitination and subsequent cellular responses. Note that XPO4 is known to function as a bidirectional nuclear transport receptor, facilitating both nuclear import and export processes.

Proper subcellular localization is essential for protein function, particularly for proteins that perform distinct roles across different cellular compartments. As E2 ubiquitin-conjugating enzymes, UBE2A/B participate in multiple biological processes, including DNA damage response, transcriptional regulation, N-end rule-mediated protein degradation, and oxidative stress responses [23,24,25,56]. Given that their major function, histone H2B K120 mono-ubiquitination, occurs in the nucleus, understanding the regulatory mechanisms governing UBE2A/B nuclear localization is of particular importance. In this study, we demonstrate that the N-terminal basic-rich region and S120 phosphorylation jointly regulate UBE2A/B nuclear localization (Figure 7).

Although proteins smaller than 40–60 kDa are generally thought to passively diffuse across the nuclear envelope, increasing evidence suggests that small proteins can also undergo active nuclear transport. For example, histone H2A–H2B heterodimers (~28 kDa) are actively imported via importin-9 and importin-4/5 [57], while histone H1 (~22 kDa) is transported by importin-β1 and importin-7 [58]. Similarly, Myc-associated factor X (~17 kDa) undergoes active nuclear import via a classical NLS-dependent mechanism [59,60]. In addition, nuclear export of small proteins can also be actively regulated, as exemplified by Exportin-1-mediated export of HINT1 [47]. Our findings further support the importance of NLS-mediated regulation even for relatively small proteins, highlighting that characterization of nuclear localization signals remains critical for understanding proteins involved in transcriptional and chromatin-related processes.

We also show that phosphorylation of UBE2A/B contributes to its nuclear localization. Although the S120A mutant did not exhibit a significant difference compared with the wild type, multiple lines of evidence—including the S120D mutant, flavopiridol treatment, structural modeling, and upstream pathway analysis—support a positive role for phosphorylation. The additional pulldown assay further showed that phosphatase inhibition enhanced the interaction between wild-type UBE2A and XPO4, whereas this effect was not observed with the S120A mutant, providing additional biochemical support for the contribution of S120 phosphorylation to the UBE2A–XPO4 interaction. The lack of phenotype in the S120A mutant may reflect compensatory mechanisms. In addition, S120 phosphorylation has been reported to regulate UBE2A/B enzymatic activity. Specifically, phosphorylation at S120 promotes RNF20/40-mediated H2B K120 mono-ubiquitination while inhibiting interaction with UBR1 and subsequent K48-linked polyubiquitin chain formation [61]. This is consistent with our previous observations that H2B mono-ubiquitination levels are altered under different mechanical conditions. Given that H2B mono-ubiquitination occurs exclusively in the nucleus, whereas N-end rule-mediated degradation can occur in both the nucleus and cytosol, differential interactions with binding partners may also contribute to the subcellular distribution of UBE2A/B. The development of a phospho-specific antibody against UBE2A S120 would provide a valuable tool for further investigating the functional role of S120 phosphorylation in UBE2A/B.

As illustrated in Figure 6B, CDK2 is a cell cycle-associated kinase that requires association with Cyclin E1/E2 or Cyclin A1/A2 for activation at different stages of the cell cycle [62,63]. Its activity can be inhibited by CDK inhibitors such as p21, p27, and p57 [64,65,66]. In addition, CDK2 activity is regulated by phosphorylation: the CAK complex (CDK7, Cyclin H, and MAT1) activates CDK2 via phosphorylation at Thr160 [67], whereas WEE1 negatively regulates CDK2 by phosphorylating Thr14 and Tyr15 [68]. This inhibition can be reversed by Cdc25A-mediated dephosphorylation [69]. In contrast, CDK9 is primarily associated with transcriptional regulation. It forms active complexes with Cyclin T1/T2 or Cyclin K [70,71], and its activity is negatively regulated by the 7SK small nuclear ribonucleoprotein (7SK snRNP) complex, which consists of 7SK snRNA, HEXIM1/2, LARP7, and MEPCE [72]. JMJD6 has been reported to cleave MEPCE, thereby releasing CDK9 from the inhibitory 7SK snRNP complex [73]. RNA-seq analysis showed that these upstream regulators for CDK2/9 respond to mechanical stimulation in a manner broadly consistent with our phenotypic observations. Nevertheless, how mechanical forces and cellular density coordinately govern these upstream factors remains poorly understood.

Taken together, these findings suggest that the upstream regulatory networks governing UBE2A S120 phosphorylation—particularly those mediated by CDK2 and CDK9—are extensively modulated by mechanosensitive cues. This model aligns with our previous observations that UBE2A/B preferentially translocate to the cytosol under conditions of high cell density, soft substrates, or blebbistatin treatment. Nonetheless, because RNA-seq data capture alterations solely at the mRNA level, they do not directly reflect actual protein activity. Whether the observed mechanosensitive changes stem from direct transcriptional regulation, alterations in mRNA stability, or feedback mechanisms originating from UBE2A/B-mediated chromatin modifications remains an open question that warrants further investigation.

Classical NLS motifs are typically short peptides enriched in basic residues [52]. Although the N-terminal region of UBE2A/B contains a high density of basic amino acids, it does not fully conform to canonical mono-partite or bi-partite NLS consensus sequences, suggesting that it may represent a non-classical NLS. This is consistent with our pulldown assay results, in which no detectable enrichment of UBE2A with KPNA1/2/3/6 was observed under the conditions tested (Figure S3). Instead, we identified XPO4 as a potential nuclear transporter of UBE2A/B. Although we were unable to directly validate the interaction between XPO4 and phosphorylated UBE2A/B in vivo, structural prediction and biochemical assays strongly support a phosphorylation-dependent interaction. Interestingly, while XPO4 has not been widely implicated in mechanotransduction, one of its known cargoes, SMAD3, is mechanosensitive [74]. SMAD3 is regulated by TGF-β signaling and exhibits complex crosstalk with the YAP1/Hippo pathway [75,76]. It can cooperate with YAP1 to regulate gene expression and cellular differentiation, while recent studies also suggest competitive interactions during nuclear import mediated by importin-7 [77]. Furthermore, alterations in XPO4 copy number and expression levels have been associated with fibrosis severity in fatty liver disease [78]. Given that fibrosis is closely linked to mechanotransduction [3,79], the potential mechanosensitivity of XPO4 and its interplay with mechanosensitive transcription factors represent important directions for future research.

## 5. Conclusions

In conclusion, we demonstrate that the N-terminal basic-rich region and S120 phosphorylation are critical determinants of UBE2A/B nuclear localization and identify XPO4 as a potential transporter mediating its nuclear import in mechanotransduction. These findings expand our understanding of UBE2A/B regulation, offering new insights into the interplay between mechanotransduction and epigenetic control. Furthermore, this regulatory axis provides a promising therapeutic target for modulating cell proliferation in CIP-deficient cancer cells.

## Data Availability

The datasets generated in this study are available from the corresponding author (F.N.; fnakamura@tju.edu.cn) upon request. Accession Codes: UBE2A: P49459; UBE2B: P63146; KPNA1: P52294; KPNA2: P52292; KPNA3: O00505; KPNA6: O60684; RAD18: Q9NS91; KPNB1: Q14974; TNPO1: Q92973; TNPO2: O14787; XPO4: Q9C0E2.

## Supplementary Materials

The following supporting information can be downloaded at: https://www.mdpi.com/article/10.3390/biology15181633/s1, Figure S1: Examples of measurement of protein-of-interest (POI)-nuclear correlation index. Figure S2: Known PTMs and NLS prediction results of UBE2A. Figure S3: KPNA1/2/3/6 do not interact with UBE2A. Figure S4: Confidence metrics of the predicted UBE2A-XPO4 complex; Figure S5: AlphaFold 3 prediction of the UBE2A/KPNA1 complex. Table S1: Primer used in this study. Table S2: Antibodies used in this study. Table S3. Quantitated source data.

## Abbreviations

UBE2A/B, ubiquitin-conjugating enzyme E2 A/B; H2B, histone H2B; H2BK120, histone H2B lysine120; ubH2BK120, ubiquitinated H2BK120; NLS, nuclear localization signals; PTM: post-translational modification; RNA-seq, RNA sequencing; KD, knock-down; NGS, next-generation sequencing; KPNA, Karyopherin Subunit Alpha; KPNB, Karyopherin Subunit beta; TNPO1/2, transportin1/2; XPO4, exportin 4.
