# Supplementary material for: N-Terminal Basic Helix and S120 Phosphorylation Cooperatively Regulate Nuclear Localization of UBE2A/B in Mechanotransduction
Source: Biology (Basel). 2026 Sep 16;15(18):1633. doi: 10.3390/biology15181633 (PMC13603920; doi:10.3390/biology15181633)

Supplementary information

N-Terminal Basic Helix and S120 Phosphorylation Cooperatively Regulate Nuclear Localization of UBE2A/B in Mechanotransduction

Mingwei Feng and Fumihiko Nakamura*

School of Pharmaceutical Science and Technology, Tianjin University, 92 Weijin Road, Nankai District, Tianjin, 300072, China.

*Corresponding author
School of Pharmaceutical Science and Technology, Tianjin University, 92 Weijin Road, Nankai District, Tianjin, 300072, China.
E-mail: fnakamura@tju.edu.cn
Phone: +86-22-87401830

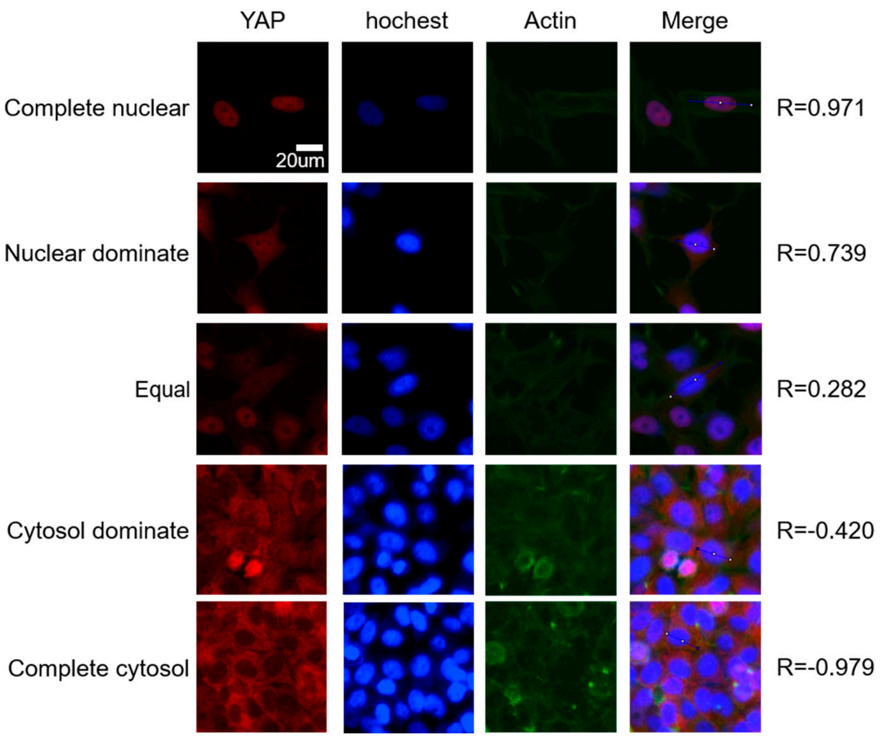

Figure S1. Examples of measurement of protein-of-interest (POI)-nuclear correlation index

hsSKM cells were cultured at different densities and stained with mouse anti-YAP1 antibody (detected with goat anti-mouse IgG secondary antibody conjugated to Alexa Fluor 594, red), phalloidin conjugated to Alexa Fluor 488 (actin, green), and nuclei with Hoechst 33342 (blue). Representative cells with nuclear YAP1 staining (top) to predominantly cytosolic YAP1 staining (bottom) are shown. The blue lines in merged images indicate the region of interest (ROI) used for correlation index (CI) calculation between YAP1 (red) and nuclear (blue) signals. Cells with primarily nuclear YAP1 exhibit positive CI values approaching 1, whereas cells with cytosolic YAP1 exhibit negative CI values approaching -1.

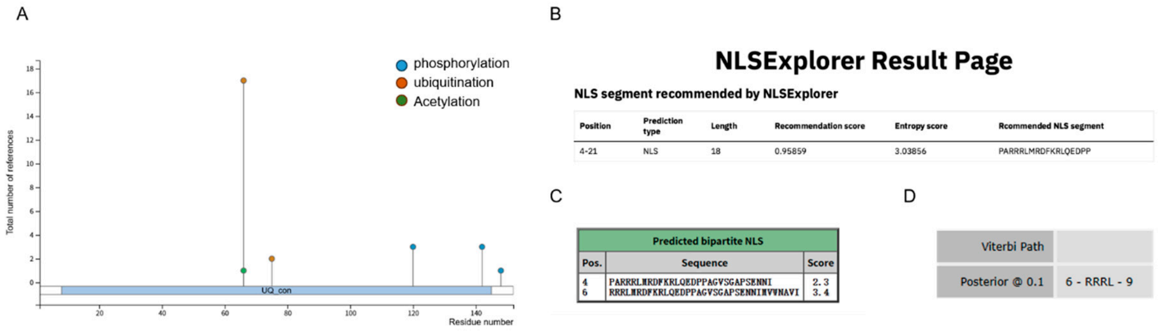

Figure S2: known PTMs and NLS prediction results of UBE2A

(A) Post-translational modification map of UBE2A obtained from PhosphoSitePlus. Phosphorylation sites are indicated in blue, ubiquitination sites in orange, and acetylation sites in green. (B) NLS prediction by NLSExplorer, indicating residues 4-21 as a potential NLS. (C) NLS prediction by NLS Mapper, indicating residues 4-39 as a potential NLS. (D) NLS prediction by NLStradamus, indicating residues 6-9 as a potential NLS.

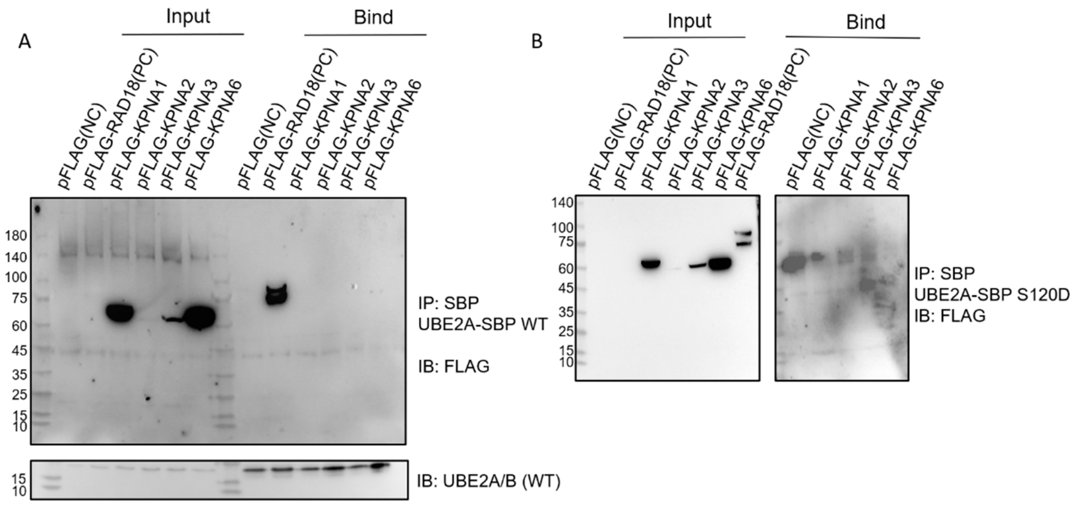

Figure S3: KPNA1/2/3/6 do not interact with UBE2A.

FLAG-tagged RAD18 (positive control), KPNA1/2/3/6, and SBP-tagged UBE2A-SBP (wild-type in panel A; S120D mutant in panel B) were expressed in HEK293A cells for 48 h. Cell lysates were incubated with NHS–streptavidin beads for pull-down. Bound FLAG-tagged importins were detected by Western blot using anti-FLAG antibody, and UBE2A-SBP was detected with mouse anti-UBE2A/B antibody. No interaction between KPNA1/2/3/6 and UBE2A-SBP was detected compared with the negative control.

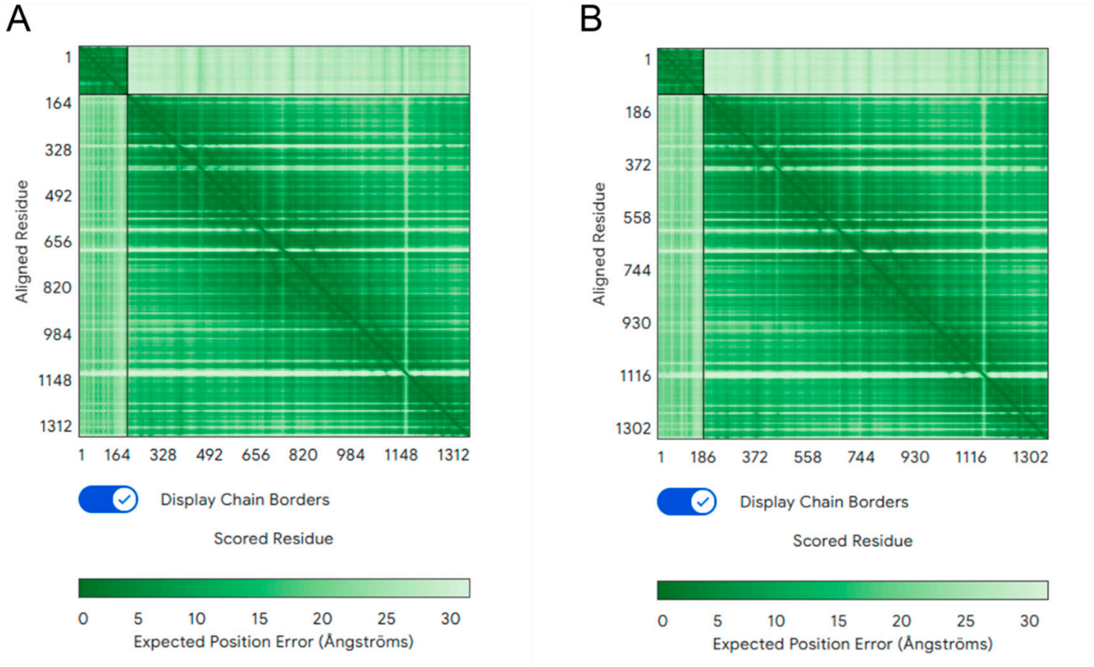

Figure S4: confidence metrics of predicted UBE2A-XPO4 complex

(A) Plots of PAE of predicted UBE2A S120pho-XPO4 complex, ipTM = 0.39 pTM = 0.75. (B) Plots of PAE of predicted UBE2A WT-XPO4 complex, ipTM = 0.37 pTM = 0.75.

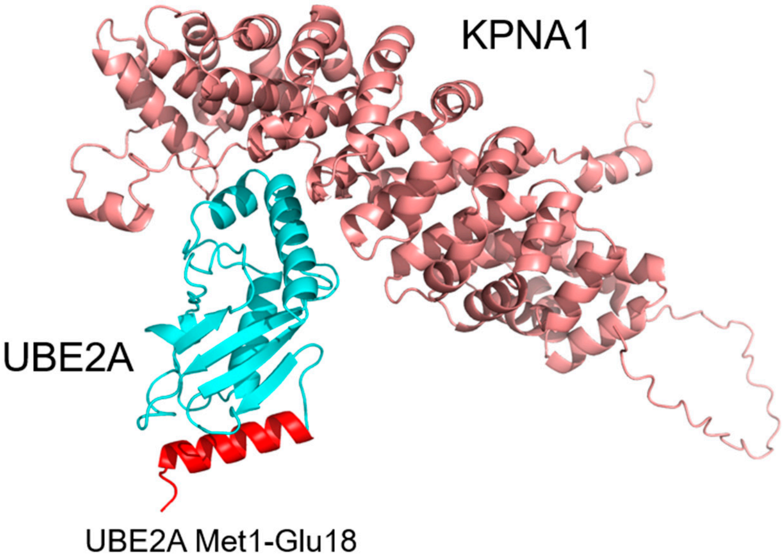

Figure S5: Alphafold3 prediction of UBE2A/KPNA1 complex.
Predicted structure of the UBE2A (cyan)-KPNA1 (salmon) complex generated using AlphaFold3. Unlike the predicted UBE2A-XPO4 complex, the N-terminal helix of UBE2A (Met1-Glu18, red) is positioned away from the KPNA1 interface, suggesting a lack of interaction.

Source image data:

Figure 3C

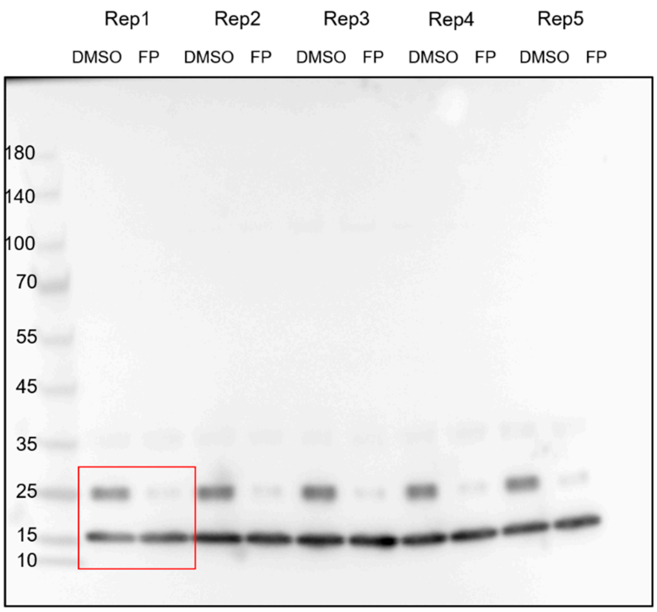

Figure 4A

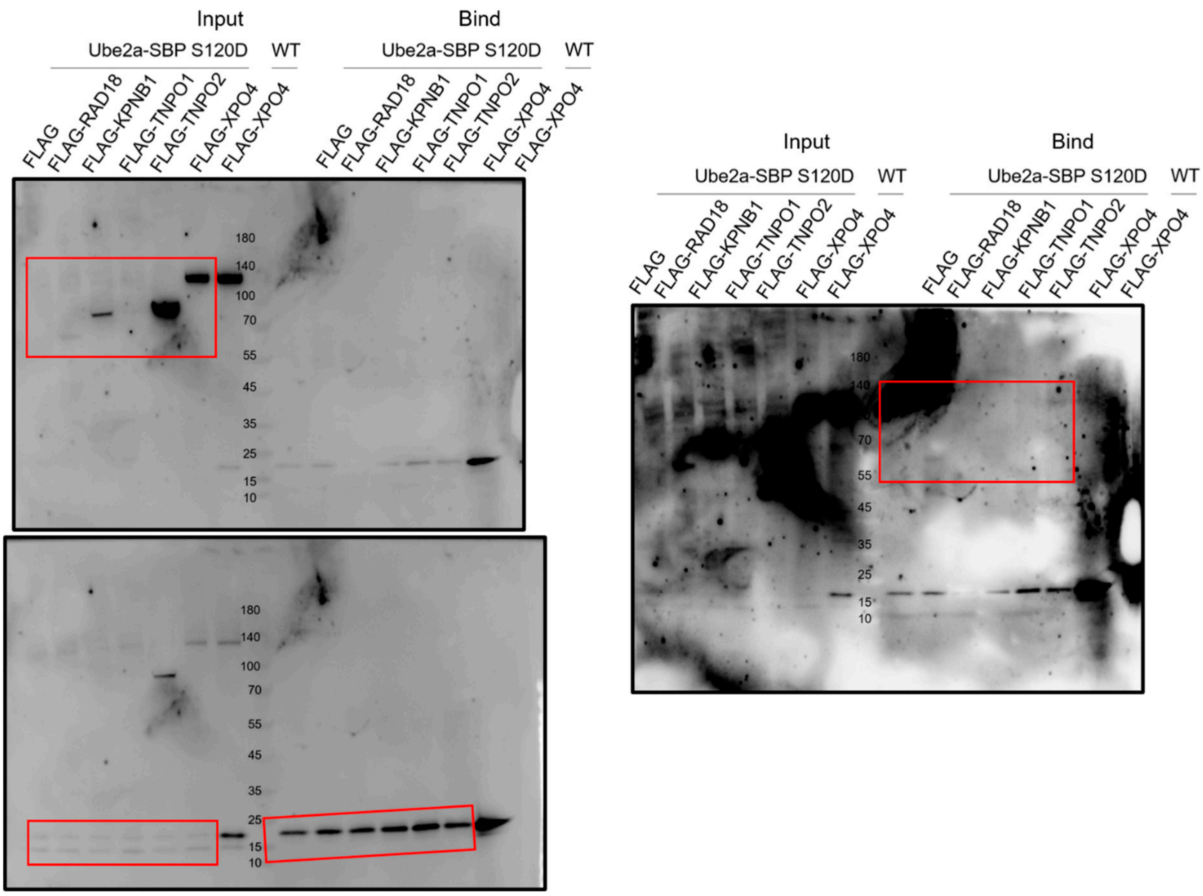
Input
Bind
Ube2a-SBP S120D
WT
FLAG
FLAG-RAD18
FLAG-KPNB1
FLAG-TNPO1
FLAG-TNPO2
FLAG-XPO4
FLAG-XPO4
180
140
100
70
55
45
35
25
15
10

Figure 4B

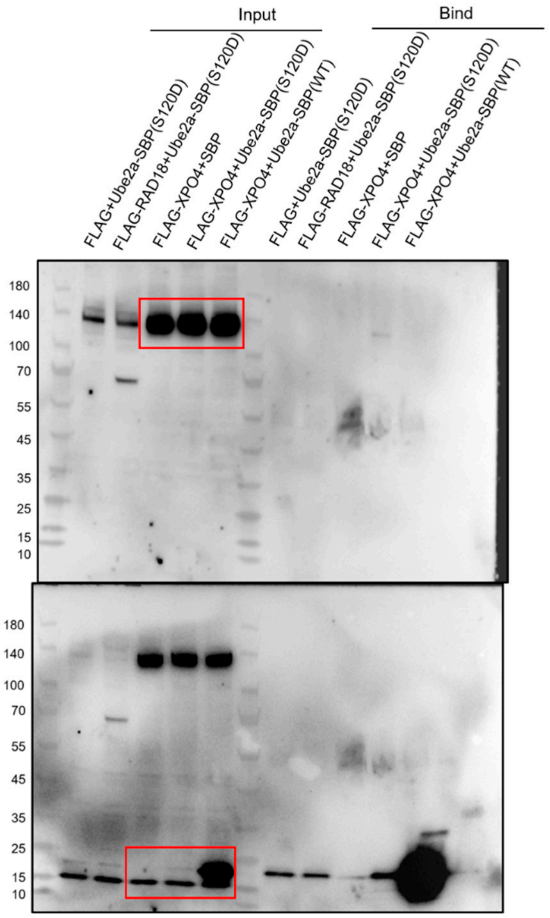

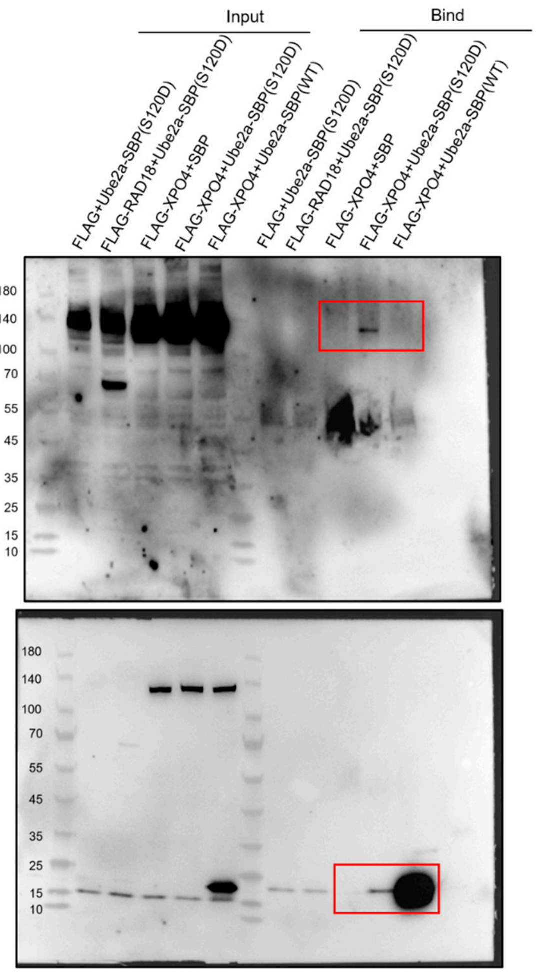

Figure 4C and 5D

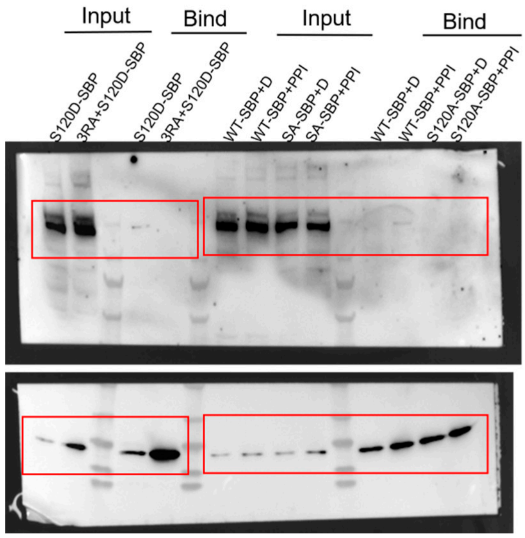

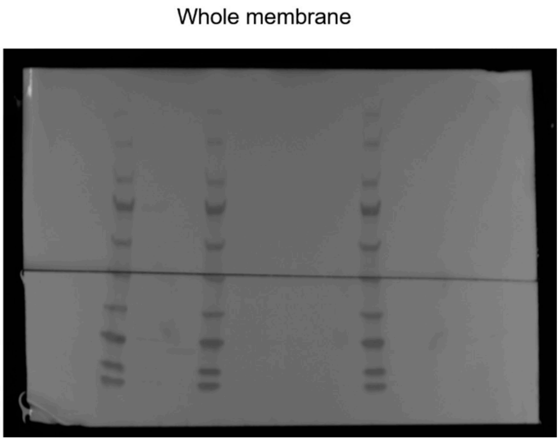

Figure S3A (bottom)

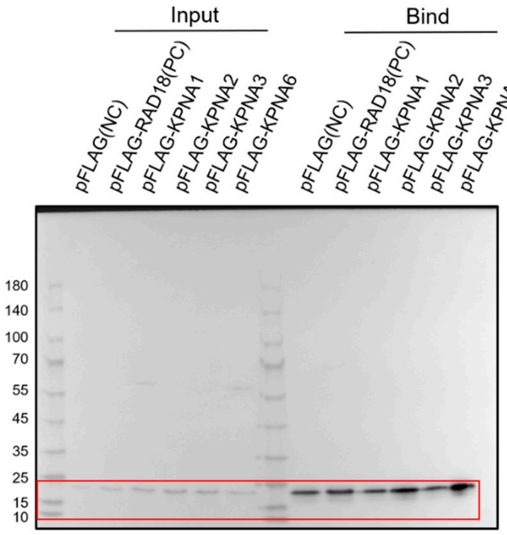

Supplement: Supplementary file 1 [file biology-15-01633-s001.zip › revised Supplementary informations.pdf]
